# The Seroprevalence and Seroincidence of Enterovirus71 Infection in Infants and Children in Ho Chi Minh City, Viet Nam

**DOI:** 10.1371/journal.pone.0021116

**Published:** 2011-07-12

**Authors:** Chau Bich Nguyen Tran, Hieu Trong Nguyen, Ha Thanh Thi Phan, Ngoc Van Tran, Bridget Wills, Jeremy Farrar, Joseph D. Santangelo, Cameron P. Simmons

**Affiliations:** 1 Oxford University Clinical Research Unit, Ho Chi Minh City, Viet Nam; 2 Centre for Tropical Medicine, University of Oxford, Oxford, United Kingdom; 3 Department of Neonatology, Hung Vuong Hospital, Ho Chi Minh City, Viet Nam; 4 Hospital for Tropical Diseases, Ho Chi Minh City, Viet Nam; 5 Inviragen Pte Ltd., Singapore, Singapore; 6 District 8 Hospital, Ho Chi Minh City, Viet Nam; Wayne State University, United States of America

## Abstract

Enterovirus 71 (EV71)-associated hand, foot and mouth disease has emerged as a serious public health problem in South East Asia over the last decade. To better understand the prevalence of EV71 infection, we determined EV71 seroprevalence and seroincidence amongst healthy infants and children in Ho Chi Minh City, Viet Nam. In a cohort of 200 newborns, 55% of cord blood samples contained EV71 neutralizing antibodies and these decayed to undetectable levels by 6 months of age in 98% of infants. The EV71 neutralizing antibody seroconversion rate was 5.6% in the first year and 14% in the second year of life. In children 5–15 yrs of age, seroprevalence of EV71 neutralizing antibodies was 84% and in cord blood it was 55%. Taken together, these data suggest EV71 force of infection is high and highlights the need for more research into its epidemiology and pathogenesis in high disease burden countries.

## Introduction

Hand, foot and mouth disease (HFMD) is a common, highly infectious childhood viral disease that can be transmitted from person to person through the fecal-oral or respiratory route. Typical clinical manifestations include a brief fever followed by pharyngitis, mouth ulcers and vesicular rash on the palms of the hands and the soles of the feet. Children may have reduced appetite due to painful vesicles in the mouth cavity. Children less than 5 years of age account for the majority of hospitalized HFMD cases [1], [2], [3], [4] and although it is usually mild and self-limiting, severe organ impairment can occur which occasionally leads to death [4], [5]. Large epidemics of HFMD in the World Health Organization's Western Pacific Region have recently been described [5] and have been associated with severe and fatal outcomes [6], [7], [8].

HFMD is caused by members of the Enterovirus genus, mainly, coxsackievirus A16 or enterovirus 71 [9]. In addition, sporadic cases with coxsackievirus types A4–A7, A9, A10, B1–B3, and B5 have been reported [2], [9]. Human enterovirus 71 (EV71) has been more frequently associated with severe forms of HFMD such as aseptic meningitis, polio-like paralysis and/or encephalitis [5]. The basis for the relatively greater virulence of EV71 is unclear but might be associated with viral genetics [10]. There are no vaccines or specific therapies to prevent or treat severe HFMD.

HFMD has emerging as a public health problem in the south of Viet Nam and is associated primarily with Coxsackievirus A16 or EV71 infection. A previous study by Tu et al. outlined the age-related epidemiology of hospitalized EV71-associated HFMD in the largest children's hospital in Ho Chi Minh City [2]. To more fully explore the scale of EV71 virus transmission in the healthy infants and children in Ho Chi Minh City, we undertook a prospective seroincidence survey of a cohort of newborn infants followed from birth to their 2^nd^ birthday. In parallel, we conducted a cross-sectional sero-prevalence study of EV71 neutralizing antibodies in Vietnamese children and adults.

## Materials and Methods

The study protocol were approved by the Scientific and Ethical committees at Hung Vuong Obstetric Hospital, Hospital for Tropical Diseases and the Oxford University Tropical Research Ethical committee. Written informed consent was obtained from the mother in the birth cohort study and the accompanying parent/guardian in the community dengue study.

### Collection of cord and infant plasma samples

The decay of maternally derived anti-EV71 neutralizing antibodies and the seroincidence of EV71 infection in the first year of life was determined using serial plasma samples collected from 200 infants born at Hung Vuong Obstetric Hospital, Ho Chi Minh City, between September 2006–September 2007. All 200 infants were participants in a prospective birth cohort, the methods for which have been described previously [11]. From each infant, cord blood was collected at the time of birth and plasma samples were collected at four follow-up visits (3, 6, 9 and 12 months).

### Collection of plasma samples in a cross-sectional survey of children and young adults

To determine the age-related seroprevalence of EV71 neutralizing antibodies in relatively older children we collected plasma from 263 young children at 12-, 18- and 24- months of age, and 120 children aged from 5–15 years. The plasma samples from infants aged 12 or 24 months of age were collected in the same birth cohort study described previously but who were not represented in the 200 infants in whom seroincidence rates were determined. The plasma samples from children aged 5–15 years were collected as part of a community-based study of dengue at District 8 Hospital, Ho Chi Minh City between September 2005–January 2009. Participants were children with suspected dengue. For the purposes of determining EV71 seroprevalence levels in this age group, we selected plasma samples from 120 children between 5 and 15 yrs of age from whom blood samples had been collected ≥14 days after study enrolment and who had no serological or virological evidence of dengue infection. The diagnosis in these “not dengue” cases was not determined, but were presumed viral infections. There was no clinical evidence to suggest these were HFMD cases. Cord blood were used to provide the results in young adults.

### EV71 virus neutralization assay

We defined neutralizing antibody levels in plasma to an EV71 virus (EV71/7423/MS87) isolated in Mississippi USA in 1987. In brief, the experiments were run in 96-well plates. Vero cells were prepared at 10^4^ cells/well and left overnight. Diluted plasma was incubated with EV71/7423/MS87 for 1.5 hours, and then incubated with plated Vero cells at 37°C, 5% CO_2_ for 5 days. The final multiplicity of infection (MOI) was 0.01. Each plasma dilution was tested in duplicate. Results were documented visually by reading the cytopathic effect (CPE) through an inverted microscope at day 5. The plasma neutralization titre was defined as the reciprocal of the dilution of plasma required to neutralize ≥50% of the virus-infected wells. An antibody titre of 20 was the limit of detection using this method.

### Statistical analysis

Statistical analyses were performed with SPSS version 14. *P*<.05 was considered to indicate statistical significance. We used the Mann Whitney U test to compare titre values between age groups. The EV71 infection incidence was calculated by observing the EV71 neutralizing-antibody (Neut-Ab) seroconversion rates among 463 infants during the first 2 years of life. We stopped counting the time that an infant contributed to the study whenever the infant showed evidence of EV71 neutralizing-antibody seroconversion, at the time of withdrawal from the study, or at the conclusion of follow-up. The incidence of EV71 infection was expressed as the number of EV71 Neut-Ab seroconversions per 100 person-years.

## Results

### Seroprevalence of virus neutralizing antibodies to EV71 in cord plasma and the decay of maternally-derived antibodies in infants

**Figure 1:**
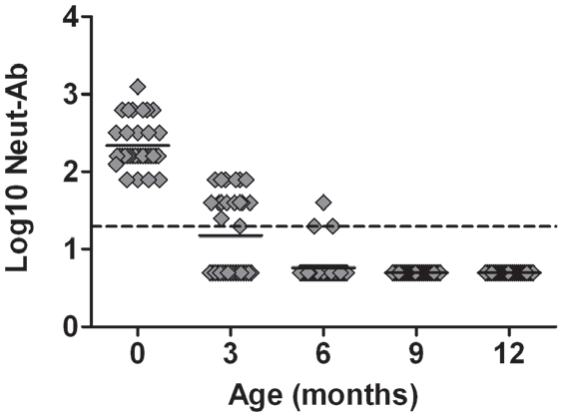
Decline of maternally-derived Neut-Ab to EV71 in 37 infants in the first year of life. The scatter plot shows Neut-Ab titres in 37 individual infants from the time of birth to 12 months. These infants all had Neut-Ab titre to Ev71 ≥80 at birth. The infant having a detectable titre at 6 months had the highest Ab titre at birth (1280). Maternal Neut-Ab to EV71 were not detected in these infants at 9 and 12 months of age. The dashed line represents the limit of detection of the assay.

Amongst 200 cord plasma samples collected during 2006–2007 from infants born in Ho Chi Minh City, 110 (55%) had detectable levels of virus neutralizing antibodies to EV71. To estimate the rate of decay of maternally-derived EV71 neutralizing antibodies, we measured their levels in serial plasma samples from infants in whom the cord blood EV71 neutralizing antibody titre was ≥80. The rationale for this was that preliminary data indicated that cord blood samples with titres ≤40, were below the limit of detection by the time the infant was 3 months of age. In the 34 infants in whom the EV71 neutralizing antibody titre was ≥80 in cord plasma, the geometric mean titre (GMT) was 220 (95% CI: 133–307). At 3 months of age, 12 (50%) of these infants still had detectable EV71 neutralizing antibody (GMT 48 (95% CI: 38–58)) whilst at 6 months of age only 2 (6%) had detectable neutralizing antibody (GMT 28 (95% CI: 9–47) (Fig. 1). Maternally-derived EV71 neutralizing antibody could not be detected in any of the infants at 9- or 12-months of age.

### Seroincidence of EV71 infection in the first two years of life

**Figure 2:**
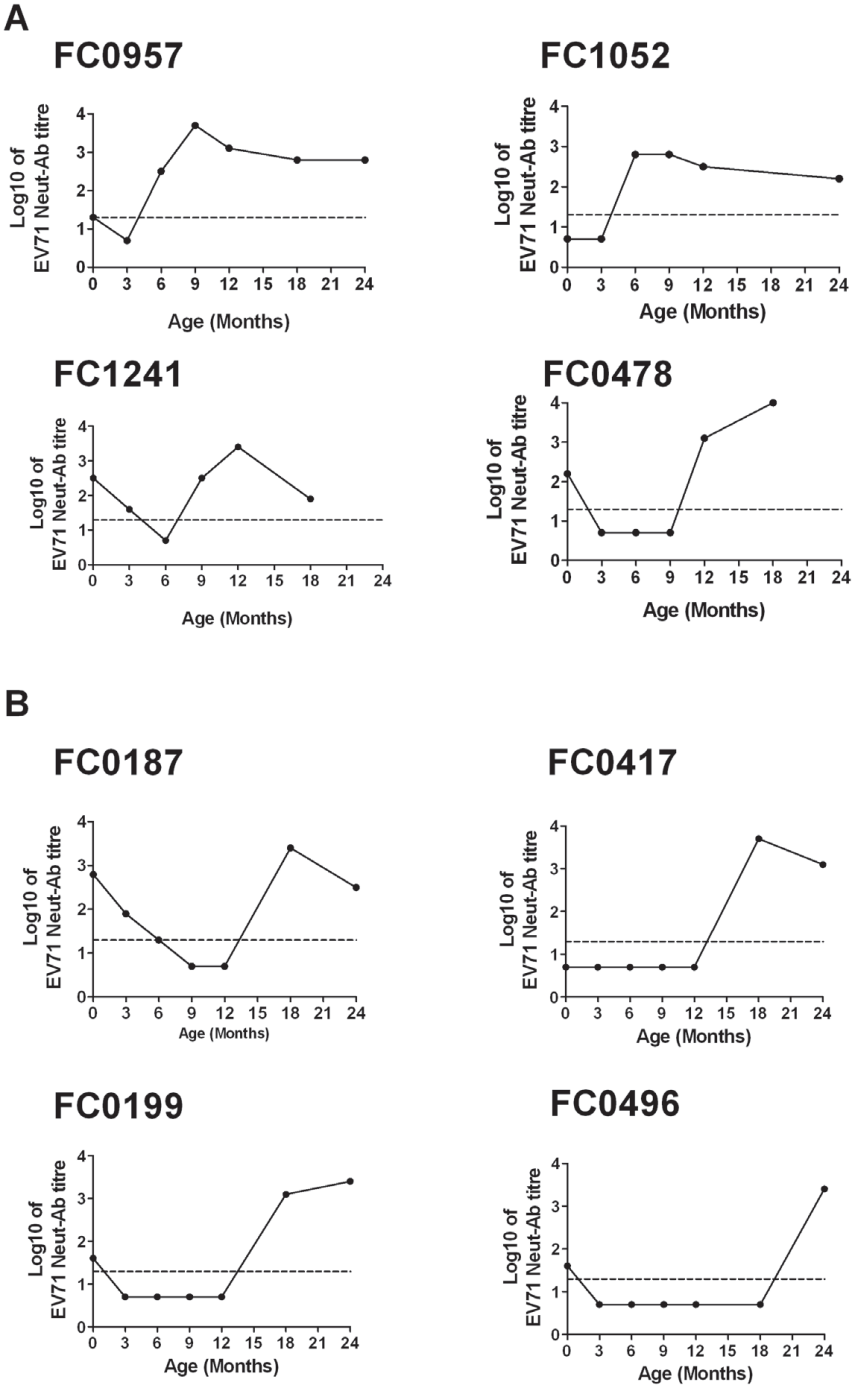
EV71 Neut-Ab profiles in 8 representative infants. Shown in panel A are representative Neut-Ab profiles from 4 infants (out of a total of 17 infants) with incident EV71 seroconversion in the first year of life. Similarly, shown in panel B are representative Neut-Ab profiles from 4 infants (out of a total of 37 infants) with incident EV71 seroconversion in the second year of life. The dashed line represents the assay limit of detection.

We used 1253 plasma samples collected between birth and two years of age from the 200 infants in whom the decay of maternal antibody was previously described and 383 plasma samples collected between 12- to 24-months of age from 263 independent young children to determine the seroincidence of EV71 infection. In the cohort of 200 infants, participants provided plasma samples every 3 months from birth to one year of age, with 42 providing a further sample at 18 months of age only, 94 providing samples at 18 and 24 months of age and 23 providing samples at 24 months of age only. The 263 independent young children provided 116, 260 and 259 plasma samples at 12, 18 and 24 month respectively. This sampling provided 6156 months of surveillance in total. EV71 infection was defined by the acquisition of virus neutralizing antibodies (seroconversion) or four-fold rise in EV71 neutralizing antibody titer. In the first year of life, 17/304 (6%) infants had evidence of EV71 infection, equivalent to an incidence rate of 5.6 per 100 person-years. The majority of these infections (12/17, 70%) occurred in months of the year previously associated with HFMD epidemics in Ho Chi Minh City (HCMC), from August to December [2]. In the 2^nd^ year of life, 37/303 (12%) infants had evidence of EV71 infection, equivalent to a rate of 14 infections per 100 person-years (Table 1). Cumulatively, by two years of age, 54/463 (11.7%) of infants in this cohort had serological evidence of EV71 infection. EV71 Neut-Ab profiles prior to and subsequent to EV71 infection in four infants infected in the first year of life (Fig. 2A) and four young children infected in the second year of life are illustrated in Fig. 2B.

**Table 1:** EV71 seroincidence in infants and young children, HCMC, Viet Nam.

Age (months)	EV71 seroconversionN (%)	No. of infants/children	Person years	EV71 infection incidence rate per 100 person-years
0–12	17 (5.6%)	304	301	5.6
12–24	37 (12.2%)	303	261.5	14

Collectively, these data suggest EV71 infection was common in this cohort of Vietnamese infants. Insight into the severity of these EV71 infections was inferred from interviews with the parents/guardians of each infant at each follow-up visit. Surprisingly, none of these infants with serologically-defined EV71 infections ever received a clinical diagnosis of HFMD at a hospital or health care provider, suggesting clinically in-apparent or very mild illness is the commonest outcome of EV71 infection in this setting.

### Seroprevalence of EV71 virus neutralizing antibodies in a cross-sectional survey of infants, children and adults

**Figure 3:**
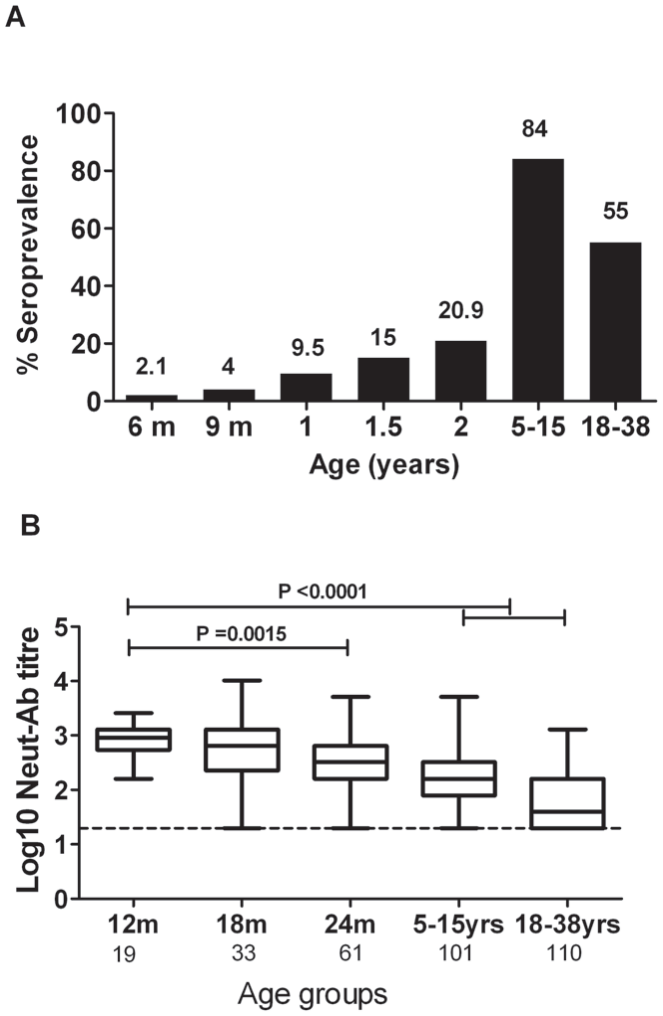
The relationship of EV71 neutralizing antibodies levels/seropositivity and age. Shown in A are age-specific seroprevalence to EV71 of Ho Chi Minh City, Viet Nam. The number above each bar shows the proportion of participants with detectable EV71 Neut-Ab in their plasma. Shown in B are levels of Neut-Ab in relation to age amongst those that are positive. The levels of Neut-Ab against EV71 in infants at 12 month-old were significantly higher than that in 24 m children (P = 0.0015), in 5–15 yrs group and 18–38 yrs group (P<0.0001) (Mann-Whitney U test). Noted that 18–38 yrs refers to cord blood. Data are shown as medians (5–95% percentile range). The dashed line represents the assay limit of detection.

To further explore the seroprevalence of EV71 virus neutralizing antibodies, we performed virus neutralization assays in plasma samples from an independent group of 275 infants aged 12 months of age, 260 infants aged 18 months of age and 259 infants aged 24 months. We complemented this assessment in infants with plasma samples from children 5–15 years of age. Consistent with the previous results, the seroprevalence at 1 year of age was 8.3%, at 18 months of age it was 13.4% and by 2 yrs of age, 23.6%. Strikingly, 84% of children aged 5–15 yrs had detectable EV71 neutralizing antibodies and there was a positive correlation between seropositivity and age (Fig. 3A). In contrast, the levels of EV71 Neut-Ab declined over age groups (Kruskal-Wallis P<0.0001) (Fig. 3B)

### Infusion of intravenous immunoglobulin (IVIG) in the treatment of severe HFMD

Infusion of intravenous immunoglobulin (IVIG) has become the standard of care for the treatment of severe HFMD in Southeast Asia [12], [13]. This treatment occurs despite the absence of any evidence from randomized controlled trials to support its use. It is plausible that IVIG preparations contain EV71-virus neutralizing antibodies that could exert an anti-viral effect in vivo. To explore this, we measured EV71-virus neutralizing antibody titres in IVIG preparations routinely used for intravenous infusion in patients with severe HFMD in Viet Nam. The two preparations, Humaglobin (Synergy Diagnostics Pvt. Ltd., Hungary) and IVIG S (GreenCross, Korean) both contained EV71-virus neutralizing antibody, with “Humaglobin” having slightly lower titres (median titre from 11 independent titrations was 438, 95% CI: 295–751)_than “IVIG S” (median titre from 3 independent titrations was 640, 95%CI: 640-640). These data indicate that IVIG preparations being used in clinical care and this setting have approximately equivalent levels of EV71-virus neutralizing antibodies and this could be relevant to the hypothesized efficacy of this treatment.

## Discussion

This study describes the decay of maternally-derived EV71 neutralising antibodies and the age-specific seroincidence and seroprevalence of EV71 in HCM City, Viet Nam between 2006 and 2009. These data contribute to our knowledge of EV71 prevalence in Southeast Asia, an important component in building the case for vaccines and therapeutics against this emerging pathogen. The incidence of EV71 infection calculated in our study was even higher than that of dengue virus infection in infants during the same period (5.6 vs 1.7 per 100 person-years) [11].

Maternally-derived neutralizing antibodies to EV71 were found to be present in 55% of cord plasma samples and these antibodies persisted for no longer than the first 6 months of life. These findings are consistent with observations made in Taiwanese infants [11], [14], [15] where the seroprevalence of Neut-Ab against EV71 decreased from 35% at birth to 1% at 6 months of age. The incidence of EV71 Neut-Ab seroconversion, which we assume to be a sensitive marker of infection, was 5.6% and 14% in the first and second year of life respectively. Strikingly, none of the infants with seroconversions were ever clinically diagnosed with HFMD. This suggests that the bulk of EV71 infections are clinically silent or very mild. Infants spend the majority of their time in the home and therefore this seems likely to be the location where EV71 infection is acquired, possibly from other members of the household. However, analysis of the baseline characteristics of the infants did not reveal any associations between seroconversion and number of siblings, socioeconomic status of the household or geographic location of residence.

That 80% of children (5–15 years) were seropositive suggests that EV71 infection is highly prevalent in Vietnamese children. Interestingly, Neut-Ab titres in children were significantly lower than infants who had recently experienced infection. This likely reflects the differences between an acute response post-infection in the infants and homeostatic levels in older children that become established months-to-years after infection. Whether these EV71 Neut-Ab titres persist for life is unknown, but since the epidemiological profile of HFMD suggests older children and adults are immune to clinical disease, it seems likely that Neut-Ab or their corresponding memory B cells persist for decades after infection occurs.

Somewhat surprisingly, the seroprevalence of EV71 Neut-Ab was higher (80%) in children than in cord blood samples (55%). Since cord blood reflects the mother's immune status, this is likely to be a reasonable estimate of the wider adult population. A 55% EV71 seropositivity rate in Vietnamese cord blood samples is consistent with that reported in previous studies from Germany (42.8%), Singapore (44%) and Taiwan (50%) [1], [3], [14]. Amongst the Vietnamese cord blood plasma samples that were positive, the titre of EV71 Neut-Ab was lower than in infants with recent infection. As observed above, this likely reflects the absence of recent EV71 exposure in adults. A possible explanation for the lower seroprevalence in adults relative to children (5–15 yrs) is true lack of exposure. The circulation of EV71 in the south of Viet Nam was first confirmed by viral culture in 2003 [2], thus it is plausible that EV71 is a relatively new introduction to southern Viet Nam. Additional seroprevalence studies and viral phylogenetics studies, as recently described for dengue in southern Viet Nam [16], could help elucidate this.

The bulk of morbidity associated with HFMD, some which is caused by EV71, occurs in infants aged 0–24 months [2], [8]. EV71 has been associated with more severe outcomes [2], [4], [17], but the pathogenic basis for this remains unknown. Since enteroviruses that cause HFMD are transmitted by fecal-oral routes, community-based efforts to encourage hand hygiene, clean water supply and sanitary conditions might be effective at limiting transmission [18], [19], [20]. Vaccination against EV71 is an attractive option. Our data, and those of others, indicates maternally derived Neut-Ab to EV71 is lost by the time an infant is 6 months of age. Were a vaccine available, the most pragmatic time to vaccinate would be when infants are 9 months of age (co-inciding with measles vaccination in many developing countries) or at 12 months of age.

There are some limitations to our study. First, the limit of detection for the neutralization assay was 20. This was established after preliminary experiments established that lower dilutions of plasma resulted in a non-specific cytopathic effects on the cell monolayer and secondly, because the volume of plasma available was insufficient to run at much lower dilutions. This limit of detection may have decreased the number of samples deemed “positive” in our study. Second, our cohorts of infants, children and adults were primarily from HCMC- different results might be observed in less urban and less developed areas of Viet Nam. We also used a genotype B EV71 virus in our neutralization assays even though the EV71 strains circulating in Viet Nam belong to genotype C4 and C5 [2], [9]. Although it is unknown if different EV71 genotypes are uniformly neutralized by “standard” polyclonal immune sera, we believe this is likely given the epidemiological evidence that recurrent episodes of EV71-associated HFMD have never been documented in the same individual.

This study provides the first report of the seroprevalence of EV71 Neut-Abs in Vietnamese infants and children. It highlighted the age-related prevalence of EV71 infection in the community of Ho Chi Minh City. Although EV71 infections were addressed in most of severe HFMD cases, our study documented that many EV71 infections are clinically silent or very mild. Therefore, further studies are needed to understand EV71 transmission and its pathogenesis in severe disease so as to inform public health interventions and clinical treatment approaches.
